# Rutin as a Mediator of Lipid Metabolism and Cellular Signaling Pathways Interactions in Fibroblasts Altered by UVA and UVB Radiation

**DOI:** 10.1155/2017/4721352

**Published:** 2017-01-12

**Authors:** Agnieszka Gęgotek, Paula Rybałtowska-Kawałko, Elżbieta Skrzydlewska

**Affiliations:** Department of Analytical Chemistry, Medical University of Bialystok, Bialystok, Poland

## Abstract

*Background.* Rutin is a natural nutraceutical that is a promising compound for the prevention of UV-induced metabolic changes in skin cells. The aim of this study was to examine the effects of rutin on redox and endocannabinoid systems, as well as proinflammatory and proapoptotic processes, in UV-irradiated fibroblasts.* Methods.* Fibroblasts exposed to UVA and UVB radiation were treated with rutin. The activities and levels of oxidants/antioxidants and endocannabinoid system components, as well as lipid, DNA, and protein oxidation products, and the proinflammatory and pro/antiapoptotic proteins expression were measured.* Results.* Rutin reduced UV-induced proinflammatory response and ROS generation and enhanced the activity/levels of antioxidants (SOD, GSH-Px, vitamin E, GSH, and Trx). Rutin also normalized UV-induced Nrf2 expression. Its biological activity prevented changes in the levels of the lipid mediators: MDA, 4-HNE, and endocannabinoids, as well as the endocannabinoid receptors CB1/2, VR1, and GPR55 expression. Furthermore, rutin prevented the protein modifications (tyrosine derivatives formation in particular) and decreased the levels of the proapoptotic markers—caspase-3 and cytochrome c.* Conclusion.* Rutin prevents UV-induced inflammation and redox imbalance at protein and transcriptional level which favors lipid, protein, and DNA protection. In consequence rutin regulates endocannabinoid system and apoptotic balance.

## 1. Introduction

Human skin plays a critical role in protecting individuals from daily exposure to external physical and chemical insults. UV radiation is the primary environmental factor that contributes to various forms of skin damage including photoaging and cancer development [1]. The UV spectrum that reaches the earth's surface contains UVB (280–320 nm) and UVA (320–400 nm) radiation. Although these two types of radiation generate different biological effects, both enhance the levels of reactive oxygen species (ROS) within cells and tissues [2]. ROS are produced physiologically during cellular metabolism and are required for cell signaling, but these molecules are also responsible for oxidative stress formation and cellular damage. UV-induced oxidative stress leads to premature skin aging by enhancing the degradation of collagen and elastin [3]. Moreover, reactive electrophiles, such as 4-hydroxyalkenals, are generated during reactions between ROS and polyunsaturated fatty acids (PUFAs) [4]. Subsequently, membrane phospholipids and proteins, including receptors, are modified by the above-mentioned electrophiles. Additional lipid mediators affected by UV-induced oxidative stress are endocannabinoids [5, 6]. They participate in cell signaling and are ligands for transmembrane receptors (mainly CB1/2 but also VR1 and GPR55); activation of CB1 is responsible for oxidative stress formation, whereas CB2 prevents ROS generation [7, 8]. However, both CB1 and CB2 stimulate the MAP kinase pathway and induce proinflammatory cascades [9].

The primary dermis cells responsible for the production of structural components, such as collagen, elastin, and glycosaminoglycans, which confer the physical and mechanical properties of the skin are fibroblasts [10]. They possess well-developed defense mechanisms against the prooxidant effects of UV radiation, including antioxidant enzymes such as superoxide dismutase, catalase, and glutathione peroxidase, as well as a number of small molecular antioxidants, such as vitamins A and E, which protect skin cells from ROS-mediated damage [11, 12]. Skin cells are also protected due to the activities of the redox-dependent transcription factors, which include Nrf2 [13]. Nrf2 is inhibited under physiological conditions by forming a complex with Keap1, but oxidative stress leads to the release, phosphorylation, and translocation of Nrf2 to the nucleus, where it binds to DNA and initiates transcription of antioxidant genes [14, 15]. UVA and UVB radiation enhance Nrf2-responsive expression of genes encoding catalase, superoxide dismutase, and antiapoptotic proteins in dermal fibroblasts [5].

**Figure 1:**
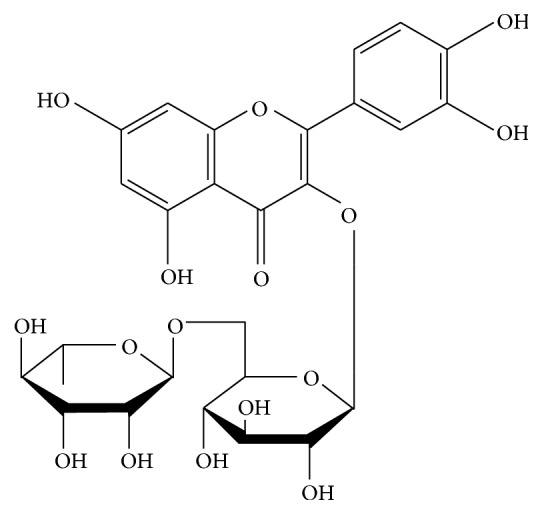
Structure of rutin.

Many natural antioxidants are used to prevent oxidative stress and its molecular consequences. One of these compounds is a plant-derived flavonoid, rutin, which is well-known nutraceutical. Rutin is present in the products of daily consumption such as buckwheat groats, vegetables, and fruits (onions, lemons) [16]. The high concentration of rutin was also reported in extracts of a number of common plants, particularly rue, barberry, or wood sorrel [17]. Rutin is a flavonol glycoside composed of quercetin and the disaccharide rutinose (Figure 1). Previous studies have indicated that rutin displays several pharmacological properties including antioxidant, anticarcinogenic, cytoprotective, antiplatelet, antithrombotic, vasoprotective, cardioprotective, and neuroprotective activities [18]. Due to their polyphenol structure, flavonoids can prevent free radical-induced injury through direct scavenging of ROS. Rutin can donate electrons to free radicals, such as hydroxyl radicals and superoxide radicals, thereby converting them into more stable, nonreactive species that terminate free radical chain reactions [18]. Rutin may also prevent oxidative stress by inhibiting the enzymes responsible for ROS generation, such as xanthine oxidase and NADPH oxidase, in rheumatoid arthritis leukocytes [19]. In addition to these direct effects on ROS levels, rutin enhances antioxidant capacity by increasing the activities of Cu, Zn-SOD, CAT, and GSH-Px and by raising GSH levels which was observed in the rat brain cells [20]. Moreover, this flavonoid inhibits the activities of cyclooxygenases and lipoxygenases, thereby reducing proinflammatory processes in human neutrophils [21]. Ischemic rats treated with rutin exhibited decreased levels of lipid peroxidation products in the kidneys [22]. Finally, rutin found in plant extracts exerts cytoprotective effects on mammalian germ cells exposed to various types of radiation by substantially increasing their viability [23].

In addition to the actions of rutin on the above-mentioned cells, rutin was also shown to exert cytoprotective effects on fibroblasts. Rutin, by lowering the levels of reactive oxygen species, decreased expression of metalloproteinases and protected skin fibroblasts against DNA modifications following exposure to UV radiation [24, 25]. By facilitating the production of extracellular matrix proteins, rutin also promoted the process of wound healing [26]. However, by modulation of cellular signaling pathways, it inhibited uncontrolled fibroblast proliferation in the myocardium [27], but whether rutin is involved in the protection of the redox balance, as well as prevention of phospholipid metabolism altered in fibroblasts due to UV radiation, is not yet known.

Therefore, the aim of this study was to examine the effects of rutin on redox and endocannabinoid systems, as well as proinflammatory and proapoptotic processes, in UV-irradiated skin fibroblasts. Interactions of electrophilic lipid peroxidation products and endocannabinoids with cellular signaling pathways after rutin administration were also examined.

## 2. Materials and Methods

### 2.1. Cell Culture and Treatment

Human fibroblasts (CCD 1112Sk) were obtained from the American Type Culture Collection. Cells were cultured in a humidified atmosphere of 5% CO_2_ at 37°C in Dulbecco's Modified Eagle Medium (DMEM) containing fetal bovine serum (10%) and supplemented with 50 U/mL penicillin and 50 *μ*g/mL streptomycin. When the cells (passages 6–8) reached 70% confluence, they were washed with PBS buffer (37°C) and exposed to UV radiation. To avoid heat stress and oxidation of the medium components, cells were exposed to UV radiation on ice in cold PBS (4°C). The exposure dose was chosen as that yielding 70% cell viability. The cells were irradiated at a distance of 15 cm from the 6 lamps (Bio-Link Crosslinker BLX 312/365; Vilber Lourmat, Germany), 6 W each, which corresponds to 4.2 mW/cm^2^ and 4.08 mW/cm^2^, respectively, for UVA (365 nm) and UVB (312 nm). Radiation doses totaled 20 J/cm^2^ and 200 mJ/cm^2^ for UVA and UVB, respectively. After receiving radiation, cells were incubated for 24 hours under standard conditions without rinsing; control cells were incubated in parallel without irradiation.

To examine the effect of rutin on UV-irradiated fibroblasts, cells were cultured in medium containing 25 *μ*M rutin (in 0.2% DMSO) [28] for 12 h before and 24 h after irradiation or only for 24 h after irradiation. Rutin used in the experiment was a purified (≥94%) natural origin commercial compound (Sigma-Aldrich, St. Louis, MO, USA). Control cells were incubated in medium containing 25 *μ*M rutin (for 24 h or 36 h) without irradiation. Changes in cell viability after UV irradiation and rutin treatment were measured using the MTT assay [29].

After treatment, all cells were washed with PBS, collected by scraping into cold PBS, and centrifuged. Cells were then resuspended in PBS and subjected to three freeze/thaw cycles. The total protein content in the cell lysates was measured using a Bradford assay [30].

### 2.2. Inflammation Processes and Intracellular ROS Generation

#### 2.2.1. Determination of Proinflammatory Factors

Proinflammatory factors TNF*α* and NF*κ*B were measured by Western blotting or by the bioimaging technique described below (*Determination of Protein Expression* and* Determination of Protein Localization*).

#### 2.2.2. Determination of Prooxidant Enzymes Activities

Xanthine oxidase (XO—EC.1.17.3.2) activity was assessed by uric acid formation from xanthine by measuring the increase in absorbance at 290 nm, according to the method of Prajda and Weber [31]. One unit of XO was defined as the amount of enzyme required to release 1 *μ*M of uric acid per minute. Analyses were performed in duplicate in three independent experiments. Enzyme specific activities were calculated in microunits per milligram of protein and expressed as a percentage of the enzyme specific activity calculated from the control cells (53.96 ± 2.98 *μ*U/mg protein).

NADPH oxidase (NOX—EC.1.6.3.1) activity was measured by luminescence assay using lucigenin as a luminophore. One unit of NOX activity was defined as the amount of enzyme required to release 1 nmol of O_2_^−^ per minute. Analyses were performed in duplicate in three independent experiments. Enzyme specific activities were calculated in RLUs (Relative Luminescence Units) per milligram protein [32] and expressed as a percentage of the enzyme specific activity calculated from the control cells (155 ± 6.78 RLU/mg protein).

#### 2.2.3. Determination of Superoxide Anions

The generation of superoxide anions was detected using an electron spin resonance (ESR) spectrometer e-scan (Noxygen GmbH/Bruker Biospin GmbH, Germany), where selective interaction of the spin probe CMH (1-hydroxy-3-methoxy-carbonyl-2,2,5,5-tetramethylpyrrolidine, 200 *μ*M) with ROS formed a stable nitroxide CM-radical with a half-life of 4 h. Thus, superoxide formation was measured from the kinetics of nitroxide accumulation according to the electron spin resonance (ESR) amplitude of the low field component of the ESR spectra [33]. Analyses were performed in duplicate in three independent experiments. The generation of superoxide anions was calculated as superoxide anion micromolar concentration per minute per milligram of protein and expressed as a percentage of the value determined from the control cells (0.035 ± 0.002 *μ*M/min/mg protein).

### 2.3. Antioxidant Defense System

#### 2.3.1. Determination of Antioxidant Enzymes Activity

Glutathione peroxidase (GSH-Px—EC.1.11.1.6) activity was assessed spectrophotometrically using the method of Paglia and Valentine [34]. GSH-Px activity was assayed by measuring the conversion of NADPH to NADP^+^. One unit of GSH-Px activity was defined as the amount of enzyme catalyzing the oxidation of 1 *μ*mol NADPH min^−1^ at 25°C and pH 7.4. Analyses were performed in duplicate in three independent experiments. Enzyme specific activity was calculated in milliunits per milligram of protein and expressed as a percentage of the enzyme specific activity determined from the control cells (10.15 ± 0.73 mU/mg protein).

Glutathione reductase (GSSG-R—EC.1.6.4.2) activity was measured according to the method of Mize and Longdon [35] by monitoring the oxidation of NADPH at 340 nm at a pH 7.4. Enzyme activity is expressed in units per milligram of protein. One unit of GSSG-R oxidized 1 mmol of NADPH/min at 25°C and pH 7.4. Analyses were performed in duplicate in three independent experiments. Enzyme specific activity was calculated in milliunits per milligram of protein and expressed as a percentage of the enzyme specific activity determined from the control cells (24.1 ± 1.2 mU/mg protein).

Superoxide dismutase (Cu/Zn-SOD—EC.1.15.1.1) activity was determined according to the method of Misra and Fridovich [36] as modified by Sykes et al. [37], which measures the activity of cytosolic SOD. One unit of SOD was defined as the amount of enzyme, which inhibits epinephrine oxidation to adrenochrome by 50%. Analyses were performed in duplicate in three independent experiments. Enzyme specific activity was calculated in milliunits per milligram of protein and expressed as a percentage of the enzyme specific activity determined from the control cells (24.5 ± 1.4 mU/mg protein).

The thioredoxin reductase (TrxR—EC.1.8.1.9) activity was measured using a commercially available kit (Sigma-Aldrich, St. Louis, MO, USA). The reaction principle on which this kit is based is the NADPH-mediated reduction of 5,5′-dithiobis(2-nitrobenzoic) acid (DTNB) to 5-thio-2-nitrobenzoic acid (TNB), which produces a strong yellow color that is measured at 412 nm [38]. Analyses were performed in duplicate in three independent experiments. Enzyme activity was measured in units per milligram of protein and expressed as a percentage of the enzyme activity determined from the control cells (12.3 ± 0.6 U/mg protein).

#### 2.3.2. Determination of Nonenzymatic Antioxidants Level

Glutathione was quantified using the capillary electrophoresis (CE) method of Maeso et al. [39]. Samples were sonicated in Eppendorf tubes with 2 mL of a mixture containing AcN/H_2_O (62.5 : 37.5, v/v) and centrifuged at 29,620*g* for 10 min. The supernatants were immediately measured by CE. Separation was performed on a 47 cm capillary (40 cm effective length) and 50 m i.d. and was operated at 27 kV with UV detection at 200 ± 10 nm. Analyses were performed in duplicate in three independent experiments. The GSH concentration was determined using a calibration curve range of 1–120 nmol/L (*r*^2^, 0.9985) and normalized for milligrams of protein. GSH concentrations are expressed as a percentage of the GSH concentration found in the control cells (13.19 ± 0.72 nmol/mg protein).

High-performance liquid chromatography (HPLC) was used to detect the level of vitamin E [40]. Briefly, cell lysates were first centrifuged at 1000 ×g for 10 min. Vitamin E was extracted from the cell lysates using hexane. The hexane phase was removed, and the remaining mixture was dried and diluted in ethanol before 50 *μ*L of it was injected onto the RP-18 column. UV detection at 294 nm was applied. The flow rate was 1 mL/min of methanol and water (95 : 5). The concentration of vitamin E was determined using a calibration curve range of 5–25 mg/L for vitamin E and was normalized for milligrams of protein. The correlation coefficient of the curve was *r*^2^ = 0.9999. Analyses were performed in duplicate in three independent experiments. The vitamin concentration is expressed as a percentage of the concentration found in the control cells (3.97 ± 0.25 *μ*g/mg protein).

Thioredoxin levels were quantified using ELISAs [41]. Prepared standards and cell lysates were diluted 10-fold in 0.05 M carbonate binding buffer (pH 9.6; 0.015 M sodium carbonate, 0.035 M sodium bicarbonate) and applied to ELISA plate wells (Nunc-Immuno MaxiSorp, Thermo Scientific, USA). Proteins were adsorbed for 5 h at 4°C. The plates were then washed with 300 *μ*L of PBS and incubated with blocking solution (5% fat-free dry milk in carbonate binding buffer) for 2.5 h at room temperature, followed by washing with 0.1% Tween 20 in PBS. The ELISA plates were then incubated at 4°C overnight with 100 *μ*L of anti-thioredoxin primary antibody per well (diluted in 1% BSA in PBS) (Abcam, Cambridge, MA, USA). After washing the wells with 0.1% Tween 20 in PBS, the plates were incubated for 30 min with peroxidase blocking solution (3% H_2_O_2_, 3% fat-free dry milk in PBS) at room temperature. Next, 100 *μ*L of goat anti-rabbit secondary antibody solution (diluted 1 : 100 in 1% BSA in PBS) (Dako, Carpinteria, CA, USA) was added to each well, and the plates were incubated for 1 h at room temperature, followed again by a washing step. Subsequently, 100 *μ*L of chromogen substrate solution (0.1 mg mL^−1^ TMB, 0.012% H_2_O_2_) in citric buffer (0.0265 M citric acid anhydrous, 0.051 M sodium hydrogen phosphate dihydrate) was added to each well, and the plates were incubated for 40 min at room temperature. The reaction was stopped by adding 50 *μ*L of 2 M sulfuric acid per well. Spectral absorption was read at 450 nm with the reference filter set to 620 nm. Analyses were performed in duplicate in three independent experiments. The thioredoxin concentration was determined using a calibration curve range of 0.5–20 *μ*g (*r*^2^, 0.9978) and was normalized for milligrams of protein. Thioredoxin levels are expressed as a percentage of the concentration found in control cells (1.37 ± 0.07 *μ*g/mg protein).

Transcription factor Nrf2 and its inhibitors and activators were determined by Western blotting and by the bioimaging techniques described below (*Determination of Protein Expression* and* Determination of Protein Localization*).

### 2.4. DNA Modifications

#### 2.4.1. Determination of 8-OHdG

8-Hydroxy-2′-deoxyguanosine (8-OHdG) was assayed by the modified LC-MS method of Dizdaroglu et al. [42]. DNA isolation was performed using a commercial kit (GenElute Mammalian Genomic DNA Miniprep Kit, Sigma, USA). The DNA concentrations in the preparations were determined spectrophotometrically, and samples were stored at −70°C until hydrolysis. DNA hydrolysis into individual nucleosides was achieved by mixing DNA samples (200 *μ*L) with 100 *μ*L of 40 mM sodium acetate/0.1 mM ZnCl_2_ (pH 5.1) and 20 *μ*L of nuclease P1 solution (20 *μ*g protein). Samples were incubated for one hour at 37°C. Thereafter, 30 *μ*L of 1 M Tris-HCl (pH 7.4) and 5 *μ*L of alkaline phosphatase solution containing 1.5 units of the enzyme were added to each sample following 1 h incubation at 37°C. All DNA hydrolysates were ultrafiltered using Ultrafree-MC filter units (cut-off of 5000 Da). 8-OHdG concentrations in hydrolysates were determined using an Agilent 1290 LC system and an Agilent 6460 triple quadrupole mass spectrometer equipped with an electrospray ionization ESI. Solvent A (0.1% formic acid in water) and solvent B (0.1% formic acid in methanol) were used in gradient mode to achieve the desired sample separations. The flow rate was set at 0.4 mL/min while the following gradient was run: 0 min, 5% solvent B; 0 to 8.0 min, 50% solvent B; 8.0 to 8.1 min, 100% solvent B; 8.01 to 12.0 min, 100% solvent B; 12.0 to 13.0 min, 5% solvent B. LC-MS/MS analysis was performed using an Agilent 1290 HPLC system interfaced with an Agilent 6560 triple quadrupole mass spectrometer with an electrospray ionization source (ESI). The samples were analyzed in the positive-ion multiple reaction monitoring (MRM) mode and the transitions of the precursors to the product ions were as follows:* m*/*z *284.1→168 (quantifier ion) and 284.1→69 (qualifier ion). The concentrations of 8-OHdG in the samples were calculated using a calibration curve range of 10–1000 pg/mL (*r*^2^ = 0.9995), which was normalized for milligrams of DNA. Analyses were performed in duplicate in three independent experiments. 8-OHdG levels are expressed as a percentage of the 8-OHdG concentration determined in control cells (7.31 ± 0.36 ng/mg DNA).

### 2.5. Lipid Metabolism

#### 2.5.1. Determination of Cyclooxygenases Activity

Cyclooxygenases 1 and 2 (COX1/2—EC.1.14.99.1) activities were measured using a commercial assay kit (Cayman Chemical Company, Ann Arbor, MI, USA), which allows for the determination of COX activities ranging from 13 to 63 nmol/min/mL. Peroxidase activity is assayed colorimetrically by monitoring the appearance of oxidized N,N,N′,N′-tetramethyl-p-phenylenediamine (TMPD) at 590 nm [43]. To distinguish COX1 activity from COX2 activity, the specific COX2 inhibitor DuP-697 was used [44]. Analyses were performed in duplicate in three independent experiments. Enzyme specific activity was calculated in nanounits per milligram of protein and expressed as a percentage of the enzyme specific activity determined from the control cells (6.2 ± 0.3 and 7.3 ± 0.4 nmol/min/mg protein for COX1 and COX2, resp.).

#### 2.5.2. Determination of Lipid Peroxidation Products

Lipid peroxidation was estimated by measuring the levels of 4-hydroxynonenal (4-HNE) and malondialdehyde (MDA). Aldehydes were measured by GC/MSMS, as the* O*-PFB-oxime-TMS derivatives, using the modified method of Luo et al. [45]. Benzaldehyde-D_6_ was added as an internal standard to the cell lysates, and aldehydes were derivatized by the addition of* O*-(2,3,4,5,6-pentafluorobenzyl)hydroxylamine hydrochloride (0.05 M in PIPES buffer, 200 *μ*L) and incubation for 60 min at room temperature. After incubation, samples were deproteinized by the addition of 1 mL of methanol, and* O*-PFB-oxime aldehyde derivatives were extracted by the addition of 2 mL of hexane. The top hexane layer was transferred into borosilicate tubes and evaporated under a stream of argon gas, followed by the addition of* N,O*-bis(trimethylsilyl)trifluoroacetamide in 1% trimethylchlorosilane. A 1 *μ*L aliquot was injected onto the column. Derivatized aldehydes were analyzed using a 7890A GC-7000 quadrupole MS/MS (Agilent Technologies, Palo Alto, CA) equipped with a HP-5 ms capillary column (0.25 mm internal diameter, 0.25 *μ*m film thickness, and 30 m length). Derivatized aldehydes were detected in selected ion monitoring (SIM) mode. The ions used were as follows:* m/z* 333.0 and 181.0 for 4-HNE-PFB-TMS,* m/z* 204.0 and 178.0 for MDA-PFB. The LOD were as follows: 4 pmol/mL for 4-HNE and 6 pmol/mL for MDA. Analyses were performed in duplicate in three independent experiments. Obtained results were normalized for milligrams of protein. 4-HNE and MDA concentrations are expressed as a percentage of the values determined for control cells (54.2 ± 2.8 and 189 ± 10 nmol/mg protein for 4-HNE and MDA, resp.).

8-Iso-prostaglandin F2*α* (8-isoPGF2*α*) was assayed by the modified LC-MS method of Coolen et al. [46] using an Agilent 1290 UPLC system interfaced with an Agilent 6460 triple quadrupole mass spectrometer with an electrospray ionization source (ESI). Briefly, samples were purified using a SEP-PAK C18 column containing octadecylsilyl silica gel. A reverse phase C18 column (2.1 mm × 150 mm, 3.5 mm) was employed. The separation was performed using a linear gradient of water (pH 5.7) and acetonitrile. 8-isoPGF2*α*–d_4_ was used as an internal standard. 8-isoPGF2*α* was analyzed in negative-ion mode using MRM mode:* m/z* 353.2→193.1 (for 8-isoPGF2*α*) and 357.2→197.1 (for 8-isoPGF2*α*-d_4_). The limit of detection (LOD) was 1 pg/mL. Analyses were performed in duplicate in three independent experiments. Obtained results were normalized for milligrams of protein. 8-isoPGF2*α* concentrations are expressed as a percentage of the concentration determined for control cells (6.2 ± 0.3 pg/mg protein).

#### 2.5.3. Determination of Endocannabinoids

Anandamide (AEA) and 2-arachidonoylglycerol (2-AG) were quantified using modified ultrahigh performance liquid chromatography-tandem mass spectrometry (UPLC-MS/MS) by the Lam et al. method [47]. Octadeuterated endocannabinoids AEA-d_8_ and 2-AG-d_8_ were added as internal standards to the cell lysates, and all cannabinoids were isolated using solid phase extraction (SPE). UPLC-MS/MS analysis was performed using an Agilent 1290 UPLC system with a Zorbax Extend C18 column (2.1 mm × 150 mm, 1.8 mm, Agilent, Santa Clara, CA, USA) and interfaced with an Agilent 6460 triple quadrupole mass spectrometer with an electrospray ionization source (ESI). The samples were analyzed in positive-ion mode using multiple reaction monitoring (MRM). Transition of the precursor to the product ion was as follows:* m*/*z *348.3→62.1 for AEA;* m*/*z *379.3→287.2 for 2-AG. The LOD were as follows: 2 pg/mL for AEA and 40 pg/mL for 2-AG. Analyses were performed in duplicate in three independent experiments. Obtained results were normalized for milligrams of protein. Endocannabinoids concentrations are expressed as a percentage of the concentrations found in control cells (16.3 ± 0.8 and 241 ± 15 fmol/mg protein for AEA and 2-AG, resp.).

### 2.6. Protein Modifications

#### 2.6.1. Determination of Structure Modifications

Protein oxidative modifications were estimated according to the levels of carbonyl groups, tryptophan, and tyrosine, as well as tyrosine derivatives. The carbonyl groups were determined spectrophotometrically (370 nm) using 2,4-dinitrophenylhydrazine [48]. The concentrations of carbonyl groups in the samples were calculated using a calibration curve (0.2–2 mmol/L, *r*^2^ = 0.9988). To detect dityrosine and tryptophan, samples were diluted in 0.1 mol/L H_2_SO_4_ (1 : 10), and fluorescence emission/excitation at 325 nm/420 nm and 288 nm/338 nm, respectively, was measured [47]. The results were normalized to fluorescence of 0.1 mg/mL quinine sulfate in 0.1 mol/L H_2_SO_4_ (Ex_325 nm_/Em_420 nm_ = 405 and Ex_288 nm_/Em_338 nm_ = 9.7), which is equivalent to 1 U of dityrosine or tryptophan, depending on the wavelength. Analyses were performed in duplicate in three independent experiments. The results were normalized for milligrams of protein and are expressed as a percentage of the values obtained for control cells (0.32 ± 0.02 U/mg protein and 16.5 ± 0.73 U/mg protein for carbonyl groups and tryptophan, resp.).

Tyrosine and its derivatives (tyrosine, 3-chlorotyrosine, and 3-nitrotyrosine) were measured by HPLC with spectrophotometric (*λ* = 280 nm) and fluorescence (Ex_280 nm_/Em_320 nm_) detection as previously described [49]. First, 10 *μ*L of freshly prepared sodium borohydride (NaBH_4_) was added to the cells. Next, samples were delipidated and relipidated by the addition of 0.3% deoxycholic acid and 50% TCA. The protein pellet was hydrolyzed at 110°C for 24 h in 1 mL of 6 M HCl and thioglycolic acid and was evaporated under nitrogen. Next, 25 *μ*L of the sample was injected into the HPLC column. Analyses were performed on an HPLC system (Agilent 1260 Infinity) with a DAD and fluorescence detector, and RP C_18_ columns (250 × 4.6 mm, 5 *μ*m) were used. The tyrosine derivatives were separated using a gradient mobile phase containing A (buffer of sodium perchlorate with H_3_PO_4_) and B [80% methanol (v/v)] as follows: 0.1 min-2% B; 20 min-2% B; 50 min-50% B; 55 min-50% B; 56 min-2% B; and 60 min-2% B. The analysis was carried out at a constant flow rate of 1 mL/min. Analyses were performed in duplicate in three independent experiments. The concentrations of tyrosine and its derivatives were determined on the basis of calibration curves prepared for the individual compounds as follows: tyrosine (75–1500 mmol/L, *r*^2^ = 0.9998), 3Cl-tyrosine (0.5–10 mmol/L, *r*^2^ = 0.9998), and 3NO_2_-tyrosine (0.25–15 mmol/L, *r*^2^ = 0.9998). Concentrations were then normalized for milligrams of protein. The results are expressed as the percentage of expression found in control cells (34.6 ± 1.7, 2.87 ± 0.16, and 0.131 ± 0.006 *μ*mol/mg protein for tyrosine, 3Cl-tyrosine, and 3NO_2_-tyrosine, resp.).

#### 2.6.2. Determination of 4-HNE-Protein Adducts

The 4-HNE-protein adducts level was measured in duplicate in three independent experiments using ELISAs [50]. 4-HNE-BSA in BSA (final BSA concentration of 10 mg/mL) was used as a standard. All samples were diluted in PBS to a protein concentration of 10 mg/mL. Prepared samples and standards were diluted 10-fold in 50 mM carbonate binding buffer (15 mM sodium carbonate, 35 mM sodium bicarbonate; pH 9.6) and added to ELISA plate wells (Nunc-Immuno MaxiSorp, Thermo Scientific, USA) at 100 *μ*L per well. Proteins were adsorbed for 5 h at 4°C. Plates were washed with 300 *μ*L of PBS and incubated with blocking solution (5% fat-free dry milk in carbonate binding buffer) for 2.5 h at room temperature, followed by a washing step (0.1% Tween 20 in PBS). The primary antibody solution (100 *μ*L of genuine anti-4-HNE-His murine monoclonal antibody, clone 4-HNE 1g4), diluted in 1% BSA in PBS, was added, and the plates were incubated at 4°C overnight. After washing the wells with 0.1% Tween 20 in PBS, the plates were incubated for 30 min with peroxidase blocking solution (3% H_2_O_2_, 3% fat-free dry milk in PBS) at room temperature. The goat anti-mouse secondary antibody solution (100 *μ*L), diluted in 1% BSA in PBS (1 : 100; Dako, Carpinteria, CA, USA), was added to the primary antibody solution mixture, and the plates were incubated for 1 h at room temperature, followed again by a washing step. Next, 100 *μ*L of chromogen substrate solution (0.1 mg mL^−1^ TMB, 0.012% H_2_O_2_) in citric buffer (0.0265 M citric acid anhydrous, 0.051 M sodium hydrogen phosphate dihydrate) was added and the plates were incubated for 40 min at room temperature. The reaction was stopped by adding 50 *μ*L of 2 M sulfuric acid. Absorption was read at 450 nm with the reference filter set to 620 nm. The concentrations of 4-HNE-protein adducts were determined using a calibration curve range of 0.5–50 pmoles/mg BSA. The correlation coefficient of the curve was *r*^2^ = 0.9987. Analyses were performed in duplicate in three independent experiments. The concentrations of 4-HNE-protein adducts are expressed as a percentage of the concentration found in control cells (6.2 ± 0.4 pmoles/mg protein).

#### 2.6.3. Determination of Protein Expression

Western blot analysis of cellular proteins was performed according to Eissa and Seada [51]. Each analysis was performed in duplicate in three independent experiments. Whole cell lysates or membrane fractions were mixed with sample loading buffer (Laemmle buffer containing 5% 2-mercaptoethanol), heated at 95°C for 10 min, and separated by 10% Tris-glycine SDS-PAGE. The same procedure was used to prepare the negative control (containing pure PBS buffer) and the positive control (commercially purchased complete cell lysate: Santa Cruz Biotechnology, Santa Cruz, CA, USA). As internal loading controls, *β*-actin and Na^+^/K^+^ ATPase (for cell lysates and membrane fractions, resp.) were used. Separated proteins in the gels were electrophoretically transferred onto nitrocellulose membranes. The blotted membranes were blocked with 5% skim milk in TBS-T buffer (5% Tween 20) for 1 h. Primary antibodies were raised against Nrf2, phospho-Nrf2 (pSer40), Keap1, TNF*α*, HO-1, Bcl-2, cyt c, p53, ERK1/2, GPR55, *β*-actin, and Na^+^/K^+^ ATPase which were purchased from Sigma-Aldrich (St. Louis, MO, USA) and used at a concentration of 1 : 1000. Bach1, KAP1, p21, p38, p62, NF*κ*B(p52), CB1, CB2, VR1, and caspases 3, 8, and 9, purchased from Santa Cruz Biotechnology (Santa Cruz, CA, USA), were also used at a concentration of 1 : 1000. Protein bands were visualized using the BCIP/NBT liquid substrate system (Sigma-Aldrich, St. Louis, MO, USA) and quantitated using the Versa Doc System and Quantity One software (Bio-Rad Laboratories Inc., CA). The results are expressed as a percentage of the expression determined in control cells.

#### 2.6.4. Determination of Protein Localization

Cells were seeded in BD Falcon™ 96-well black, clear-bottom tissue culture plates at 10,000 cells per well. These plates are optimized for imaging applications. Analyses were performed in duplicate in three independent experiments. After incubation, cells were rinsed with PBS and fixed with a 3.7% formaldehyde solution at room temperature for 10 min. Cells were then washed three times with PBS and permeabilized with 0.1% Triton X-100 at room temperature for 5 min. Next, the cells were washed twice with PBS, and nonspecific binding was blocked by incubation in 3% FBS at room temperature for 30 min. The cells were rinsed and incubated with either anti-Nrf2 rabbit polyclonal antibodies (Sigma-Aldrich, St. Louis, MO, USA; 1 : 1000) or anti-NF*κ*B (p52) mouse polyclonal antibodies (Santa Cruz Biotechnology, Santa Cruz, CA, USA) for 1 h at room temperature. Cells were then washed three times with PBS and incubated with FITC-conjugated anti-rabbit secondary antibodies (BD Pharmingen, San Diego, CA) for 60 min in the dark. After washing, nuclei were stained with Hoechst 33342 (2 *μ*g/mL) and analyzed using a BD Pathway 855 confocal microscope with a 40x (0.75 NA) objective. The cytoplasmic and nuclear fluorescence intensities of stained cells were analyzed, and images of FITC-labeled cells were acquired using a 488/10 excitation laser and a 515LP emission laser.

### 2.7. Statistical Analysis

Data were analyzed using standard statistical analysis methods, including one-way Student's *t*-test for multiple comparisons, and the results are expressed as the mean ± standard deviation (SD) for *n* = 3. *p* values less than 0.05 were considered statistically significant.

## 3. Results

These experiments were performed using control cells, UVA- and UVB-irradiated cells, cells treated with rutin only after irradiation, and cells treated with rutin before and after UV exposure. However, due to a lack of statistically significant changes for most parameters in rutin-pretreated cells compared to treated cells, these results were omitted in the following descriptions. Consequently, the figures primarily show results from the cells treated with rutin only after irradiation.

### 3.1. Inflammatory and Oxidative Processes

**Figure 2:**
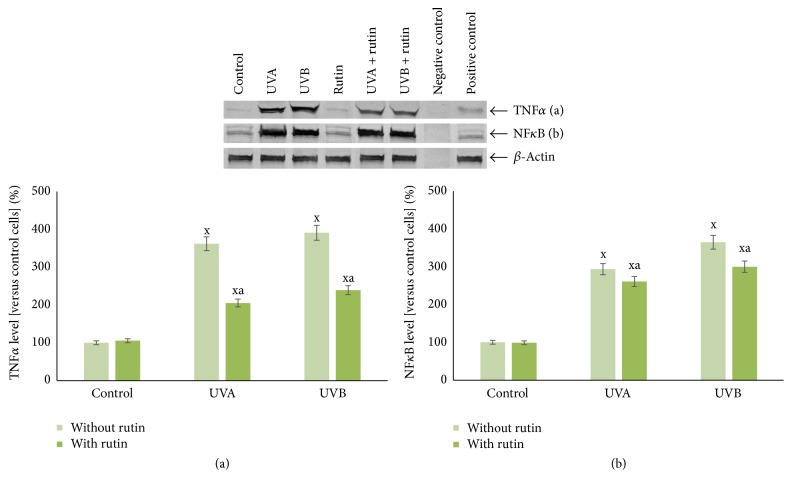
The level of proinflammatory factors TNF*α* (a) and NF*κ*B (b) in fibroblasts after exposure to UVA [20 J/cm^2^], UVB radiation [200 mJ/cm^2^], and rutin [25 *μ*M] treatment, expressed as a percentage of the control cells value. Mean values ± SD of five independent experiments are presented. ^x^Statistically significant differences versus control group, *p* < 0.05. ^a^Statistically significant differences versus group without rutin, *p* < 0.05.

**Figure 3:**
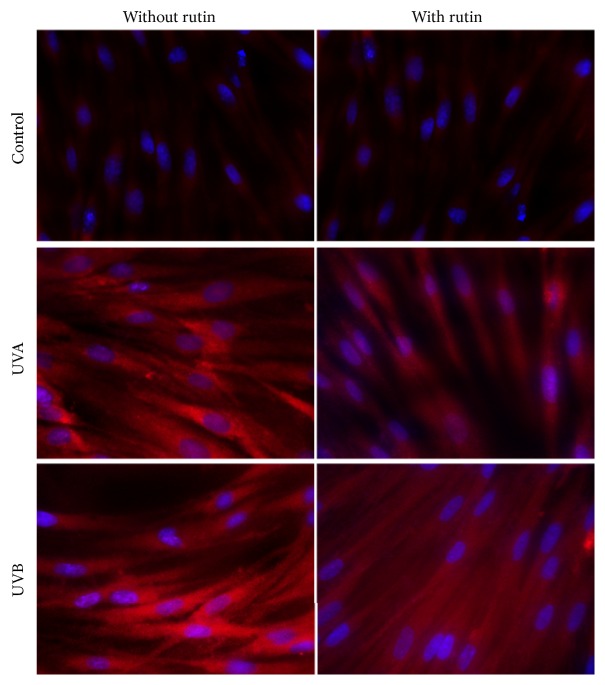
The cytoplasmic and nucleus level of NF*κ*B in fibroblasts control cells and after exposure of UVA [20 J/cm^2^], UVB radiation [200 mJ/cm^2^], and rutin [25 *μ*M] (blue, nucleus; red, NF*κ*B).

Rutin partially protected fibroblasts against expression of proinflammatory signaling mediators and intracellular oxidative processes enhanced by UVA and UVB radiation. Treatment of cells with rutin reduced NF*κ*B levels, which had been increased 3- to 4-fold after UV irradiation, by approximately 10% and 20%, respectively. TNF*α* levels were also reduced by approximately 40% after a 4-fold increase following UVA and UVB exposure (Figure 2). Moreover, rutin reduced the translocation of NF*κ*B from the cytoplasm to the nucleus, which had been enhanced by UVA and UVB exposure to varying degrees (Figure 3).

**Figure 4:**
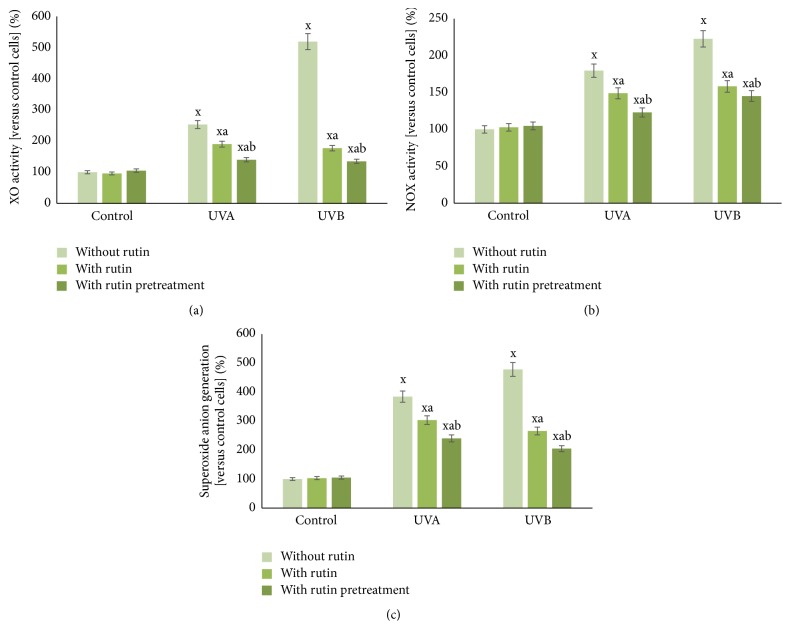
The xanthine oxidase activity (a), NADPH oxidase activity (b), and superoxide anion generation (c) in fibroblasts after exposure to UVA [20 J/cm^2^], UVB radiation [200 mJ/cm^2^], and rutin [25 *μ*M] treatment and pretreatment, expressed as a percentage of the control cells value. Mean values ± SD of five independent experiments are presented. ^x^Statistically significant differences versus control group, *p* < 0.05. ^a^Statistically significant differences versus group without rutin, *p* < 0.05. ^b^Statistically significant differences versus group without rutin pretreatment, *p* < 0.05.

Rutin significantly prevented (by approximately 20–30%) an increase in the activity of the main enzymes responsible for superoxide anion generation (xanthine oxidase and NADPH oxidase), which was increased as a consequence of UV irradiation. UVA and UVB irradiation resulted in an approximately 3- and 5-fold increase in the activity of xanthine oxidase and 80% and 120% increase in NADPH oxidase activity (Figure 4). As a result, the 4- and 5-fold increases (after UVA and UVB irradiation) in the levels of superoxide anions were reduced to 3- and 2.5-fold, respectively, by rutin treatment.

### 3.2. Antioxidant Defense System

**Figure 5:**
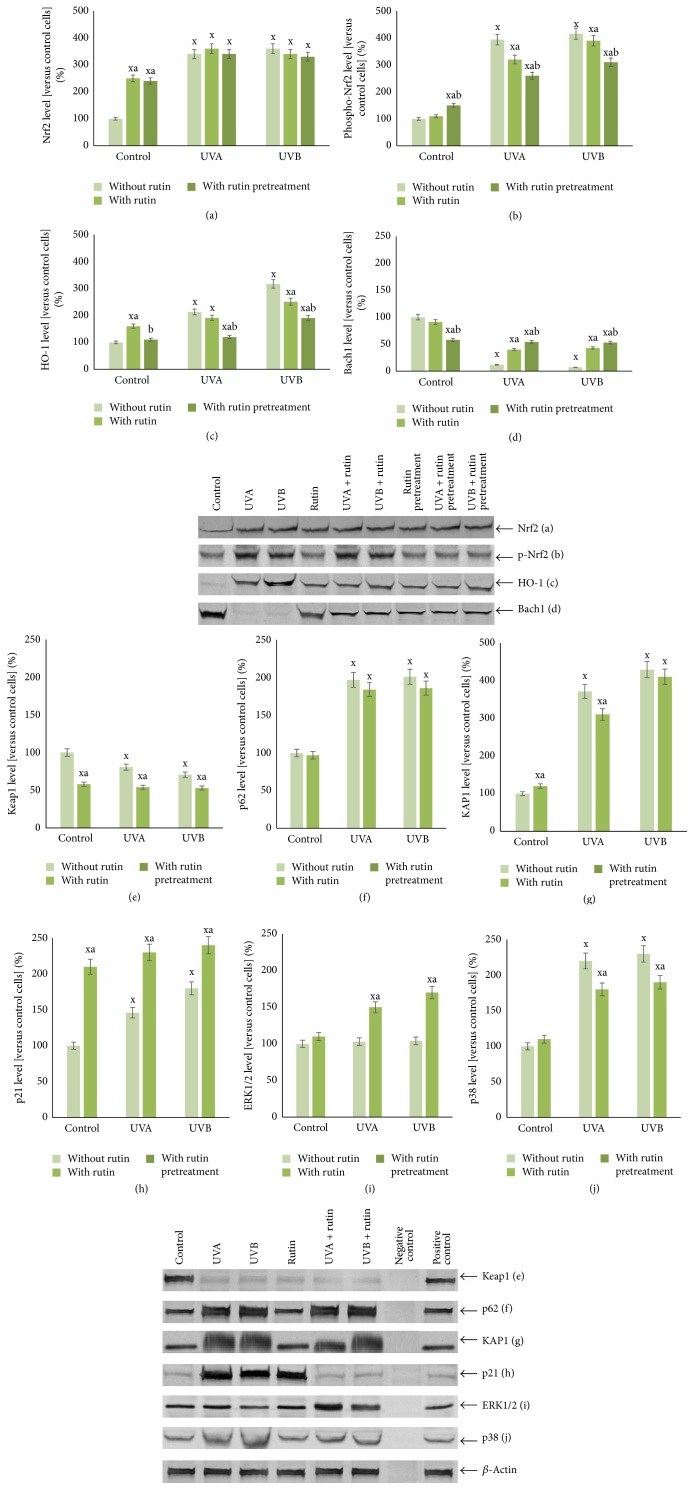
The level of Nrf2 (a) and phospho-Nrf2 (b), its main target, HO-1 (c), its inhibitors (Bach1 (d), Keap1 (e)), and activators (p21, p62, KAP1, ERK1/2, and p38 (f–j)) in fibroblasts after exposure to UVA [20 J/cm^2^], UVB radiation [200 mJ/cm^2^], and rutin [25 *μ*M] treatment and pretreatment, expressed as a percentage of the control cells value. Mean values ± SD of five independent experiments are presented. ^x^Statistically significant differences versus control group, *p* < 0.05. ^a^Statistically significant differences versus group without rutin, *p* < 0.05. ^b^Statistically significant differences versus group without rutin pretreatment, *p* < 0.05.

**Figure 6:**
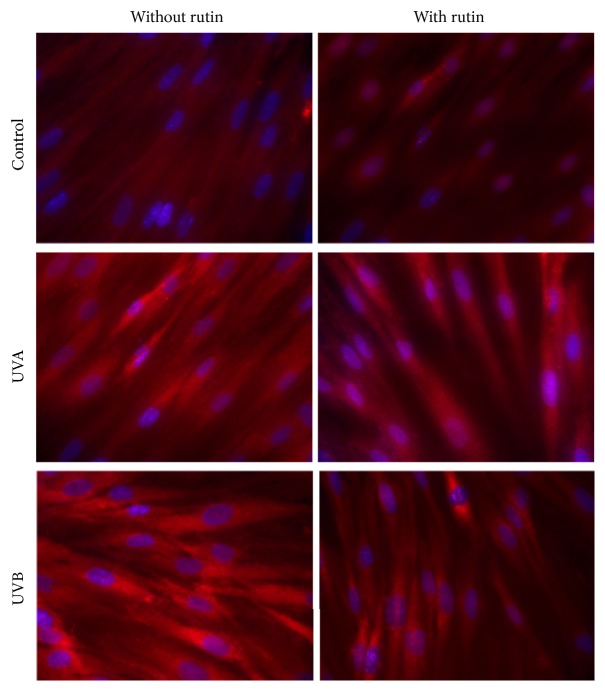
The cytoplasmic and nucleus level of Nrf2 in fibroblasts control cells and after exposure of UVA [20 J/cm^2^], UVB radiation [200 mJ/cm^2^], and rutin [25 *μ*M] (blue, nucleus; red, Nrf2).

Rutin reduced UVA-induced phospho-Nrf2 and UVB-induced HO-1 expression in fibroblasts by approximately 15% (Figure 5) and reduced UV-induced Nrf2 translocation from the cytoplasm to the nucleus (Figure 6). Moreover, rutin counteracted UV-induced changes in Nrf2 inhibitors, causing an approximately 30% increase in the Bach1 and an approximately 10% and 30% decrease in the Keap1 compared to that observed after UVA and UVB irradiation without treatment. Moreover, rutin induces an approximately 70% increase in the expression of Nrf2 activator p21 compared to that observed after UVA and UVB irradiation without treatment. However, rutin does not cause statistically significant changes in the level of other Nrf2 activators KAP1 and p62. Furthermore, rutin reduced p38 levels by approximately 20% in comparison to irradiated cells, which led to 50% and 70% increases in ERK1/2 levels in UVA- and UVB-irradiated fibroblasts, respectively (Figure 5).

In addition to transcriptional alterations, changes in the activities of antioxidant enzymes, as well as the level of nonenzymatic antioxidants, were observed in cells treated with rutin following UVA and UVB irradiation (Table 1). Rutin ameliorated UVA- and UVB-induced decreases in Cu/Zn-SOD activity by 15% and 50%, respectively. Moreover, treatment of UV-irradiated cells with rutin decreased GSH-Px activity by 20% and 45%, respectively, compared to UV-irradiated cells not treated with rutin. With respect to GSSG-R activity, rutin treatment resulted in a 16% decrease compared to UVA-irradiated fibroblasts, where approximately 2-fold increases were observed. Rutin also attenuated decreases in GSH (by 22% and 31%) and vitamin E levels (by 10% and 15%) compared to UVA- and UVB-treated cells. Furthermore, rutin treatment prevented a reduction in the thioredoxin system caused by UVA and UVB irradiation; the thioredoxin level and thioredoxin reductase activity were higher by approximately 20% and 30%, respectively (Table 1).

### 3.3. Oxidative Modifications of Cellular Components

#### 3.3.1. Lipid Mediators

**Figure 7:**
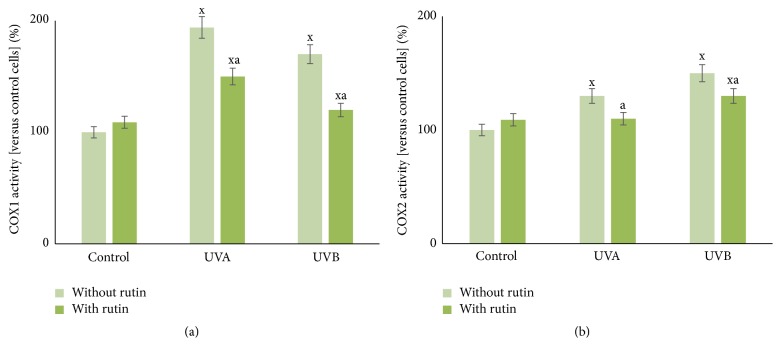
The cyclooxygenases 1 and 2 (COX1 (a), COX2 (b)) activities in fibroblasts after exposure to UVA [20 J/cm^2^], UVB radiation [200 mJ/cm^2^], and rutin [25 *μ*M] treatment, expressed as a percentage of the control cells value. Mean values ± SD of five independent experiments are presented. ^x^Statistically significant differences versus control group, *p* < 0.05. ^a^Statistically significant differences versus group without rutin, *p* < 0.05.

In fibroblasts exposed to UVA and UVB radiation, rutin prevented UV-induced increases in the activities COX1 and COX2, which are responsible for metabolism of arachidonic acid and the formation of prostanoids. Rutin reduced the activity of both of these enzymes by approximately 10% (Figure 7). Rutin treatment also protected UV-irradiated cells against increased levels of phospholipid peroxidation products. Rutin reduced the levels of 4-HNE by 25% and 27% and the levels of MDA by 40% and 30%, respectively, compared to cells exposed to UVA and UVB without the addition of rutin. Additionally, rutin decreased the levels of the iso-prostaglandin F2*α* by approximately 30% in UVA-exposed cells (Table 2).

**Figure 8:**
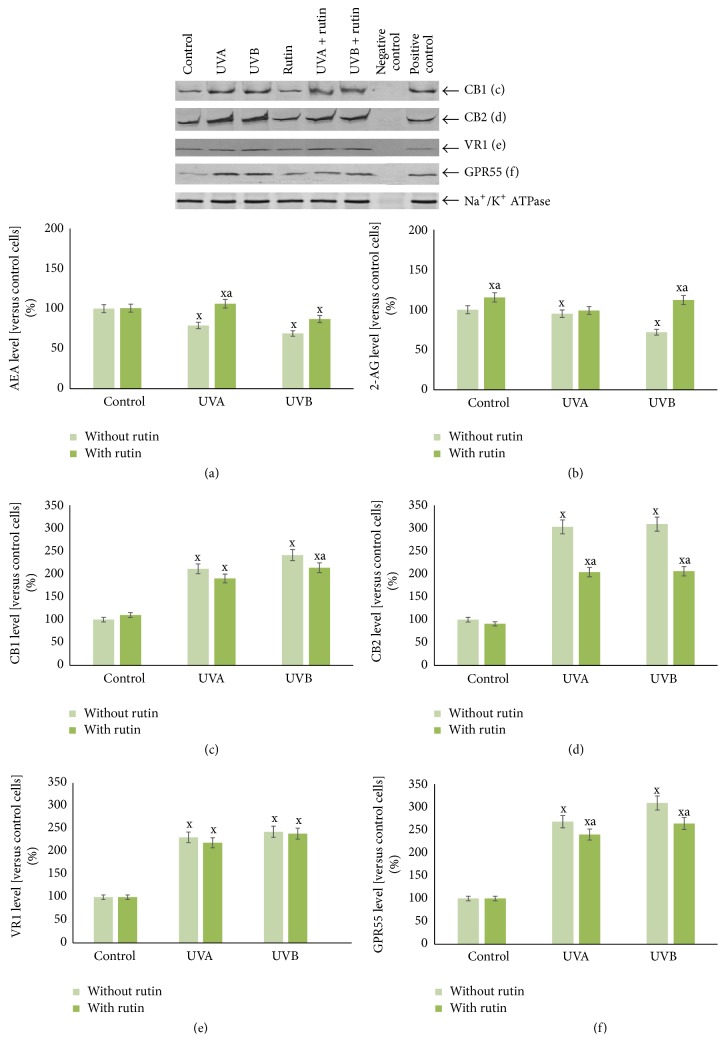
The level of endocannabinoids (AEA (a), 2-AG (b)) and their receptors (CB1, CB2, VR1, and GPR55 (c–f)) in fibroblasts after exposure to UVA [20 J/cm^2^], UVB radiation [200 mJ/cm^2^], and rutin [25 *μ*M] treatment, expressed as a percentage of the control cells value. Mean values ± SD of five independent experiments are presented. ^x^Statistically significant differences versus control group, *p* < 0.05. ^a^Statistically significant differences versus group without rutin, *p* < 0.05.

Changes in the fibroblasts' endocannabinoid system caused by UV irradiation were prevented by addition of rutin. Rutin increased AEA levels approximately 35% and 40% compared to levels in UVA- and UVB-irradiated cells not treated with rutin, respectively. It also increased the levels of 2-AG by 50% compared to UVB-irradiated cells lacking rutin. Furthermore, rutin decreased the expression of two endocannabinoid receptors (CB2 and GPR55) by approximately 30% and 15% in UVA- and UVB-exposed cells, respectively; UVA and UVB irradiation alone enhanced expression 2- and 3-fold, respectively (Figure 8).

#### 3.3.2. Protein Modifications

*(i) Structural Changes*. Rutin protected protein functions within fibroblasts through the prevention of structural modifications after UV irradiation. It reduced the levels of carbonyl groups by 5% (UVA) and 20% (UVB), which had increased after UVA and UVB irradiation by 50% and 90%, respectively (Table 2). Moreover, rutin completely abolished UV-induced increases in 3Cl-tyrosine, yet only reduced 3NO_2_-tyrosine and dityrosine levels by approximately 10–15% and increased tryptophan levels by approximately 10% (Table 2). Additionally, as a result of decreased 4-HNE levels after rutin treatment, we observed a significant decrease (approximately 20% for both types of radiation) in the formation of 4-HNE-protein adducts (Table 2). 

**Figure 9:**
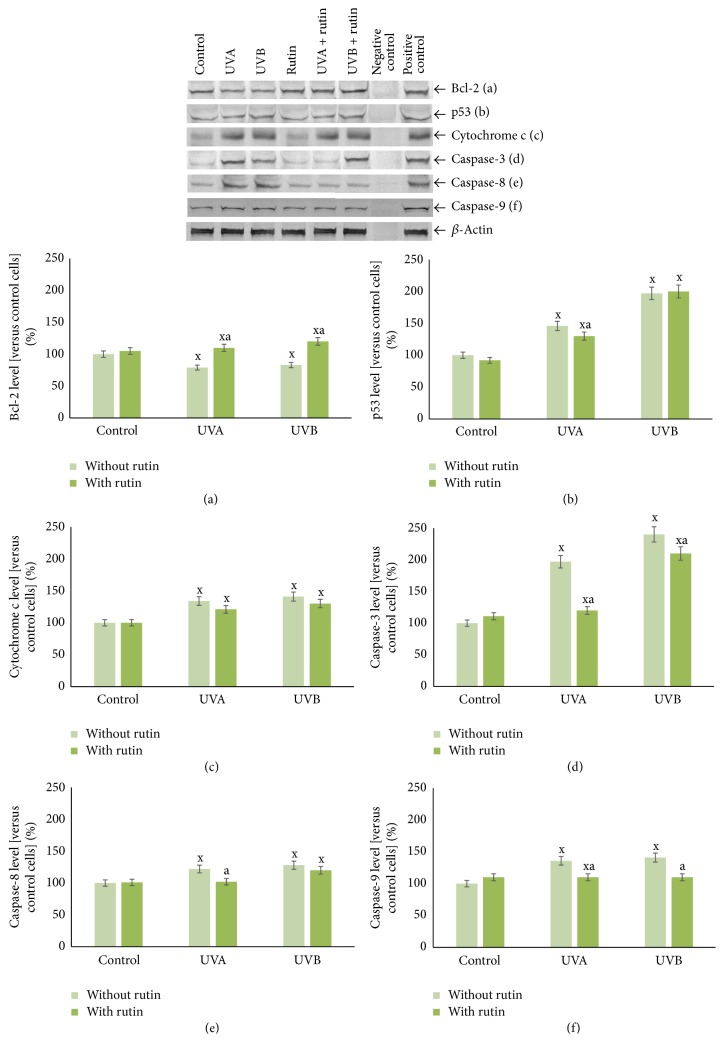
The level of anti- and proapoptotic proteins (Bcl-2 (a), p53 (b), and cytochrome c (c)) and executive caspases (3, 8, and 9 (d–f)) in fibroblasts after exposure to UVA [20 J/cm^2^], UVB radiation [200 mJ/cm^2^], and rutin [25 *μ*M] treatment, expressed as a percentage of the control cells value. Mean values ± SD of five independent experiments are presented. ^x^Statistically significant differences versus control group, *p* < 0.05. ^a^Statistically significant differences versus group without rutin, *p* < 0.05.

*(ii) Apoptotic Balance*. Rutin affected the expression levels of proteins involved in apoptosis in UV-irradiated fibroblasts through increased Bcl-2 expression, by approximately 30% and 100%, in UVA- and UVB-irradiated cells, respectively. Additionally, we observed slight decreases in the levels of cytochrome c and caspase-8, as well as a 20% reduction in caspase-9 expression. Finally, after rutin treatment, caspase-3 levels were strongly reduced in UVA- and UVB-irradiated cells by 40% and 15%, respectively (Figure 9).

#### 3.3.3. DNA Modifications

Rutin treatment protected DNA against UV-induced oxidative damage. In UVA- and UVB-irradiated fibroblasts not treated with rutin, increased levels of 8-OHdG (28% and 67%, resp.) compared to control cells were observed, while in UV-irradiated cells treated with rutin there was a greater than 10% reduction in the level of 8-OHdG (Table 2).

In addition to the metabolic responses observed in fibroblasts treated with rutin after irradiation, pretreatment of fibroblasts with rutin prior to irradiation also invoked certain notable cellular responses. Rutin pretreatment, in particular, resulted in statistically significant decreases in the activities of XO and NOX, as well as in superoxide anion generation, compared to cells treated with rutin only after irradiation. Moreover, rutin pretreatment more robustly prevented increases in Nrf2 and HO-1 expression and reduced the levels of Bach1. These changes were accompanied by reduced levels of protein oxidation markers such as carbonyl groups.

## 4. Discussion

Information pertaining to the detrimental effects of UVA and UVB radiation on human skin has been increasing [1, 13]. Therefore, there is a significant need for natural compounds that could effectively protect human skin from solar radiation. Flavonoids, including rutin, represent a promising group of nutraceuticals that are being investigated as protective agents against different environmental insults [23, 24, 52]. Rutin significantly enhances the proliferation of skin fibroblasts in rat dorsal wounds and the synthesis and accumulation of extracellular matrix components, including collagen and fibronectin, following mechanical injury, and inhibits the formation of fibrils in APPswe mouse cells [3, 26, 53]. In the present study, the molecular mechanisms involved in rutin's protection of skin fibroblasts against UVA and UVB radiation were investigated.

### 4.1. Rutin Decreases UV-Induced Inflammation

This study showed that rutin partially protected skin fibroblasts against UVA- and UVB-mediated inflammatory response. Rutin diminished levels of NF*κ*B and products of its transcriptional activity, such as TNF*α*, in UV-irradiated fibroblasts. NF*κ*B levels are dependent on prostaglandins [54]; therefore, diminished levels of prostaglandin derivatives, such as F_2*α*_ isoprostanes, observed in these studies after rutin treatment, may lead to decreased NF*κ*B levels. Decreases in this proinflammatory factor were also observed in various tissues of rutin-treated rats and were accompanied by secretion of proinflammatory cytokines after LPO-induced inflammation [55, 56]. Studies have suggested that rutin suppresses phosphorylation of NF*κ*B via inhibition of MAPK in lung tissue, in addition to reducing the expression and cytoplasmic relocation of NF*κ*B [56]. Changes observed in UV-irradiated fibroblasts treated with rutin showed lower activity of cyclooxygenases, key enzymes in the inflammatory process, which were enhanced by UV exposure. Rutin has been shown to exert anti-inflammatory effects in UVB-irradiated mouse skin by inhibiting COX-2 and iNOS expression via suppression of p38/MAPK [57]. Our results also indicated that rutin suppresses p38 levels, confirming these previous studies. Inhibition of NF*κ*B activity may also be connected with decreases in fibroblast endocannabinoids level [58]. In fact, the overexpression of cannabinoid receptors in response to UV irradiation and the subsequent decrease following rutin treatment did not correlate with changes in endocannabinoids level. Because the expression of all examined cannabinoid receptors was enhanced following UV irradiation, it is likely that different, albeit undefined, mechanisms/agonists mediated their activation in this context. Previous data indicated that CB1/2 receptors play a key role in UV-induced skin inflammation [9]. The results of the study presented here showed that rutin partially blocks UV-induced activation of cannabinoid receptors and has particularly robust effects on CB2. The expression of endocannabinoids and their receptors following UV irradiation and rutin treatment may also be associated with the actions of enhanced F_2*α*_ isoprostanes that may act as potent cannabinoid receptor ligands, thus causing their activation [59, 60].

### 4.2. Rutin Prevents Intracellular ROS Generation after UV Irradiation

Previous report and data from the study presented here demonstrated that UV radiation perturbs the fibroblast redox balance by enhancing the activity of ROS-generating enzymes [5]. Enhanced activity of xanthine and NADPH oxidases, the primary cellular enzymes responsible for the generation of superoxide radicals, is attenuated by rutin. Like many other flavonoids, rutin can scavenge free radicals and chelate transition metal ions, which participate in Fenton reactions to generate reactive hydroxyl radicals, results that can be attributed to its polyphenolic structure [18, 61, 62]. The main functional groups in the rutin molecule responsible for its antioxidant activity are the hydroxyl groups at positions 5 and 7 of the A ring, as well as the double bond in the C ring of the quercetin-polyphenolic component [63]. Moreover, it was previously shown that rutin could inhibit the overproduction of oxygen radicals by neutrophils under pathological conditions such as rheumatoid arthritis or cancer [19, 64].

### 4.3. Rutin Contributes to Antioxidant Defenses at the Transcriptional Level after UV Irradiation

In addition to its direct effect on ROS generation, rutin also protected fibroblasts against UV damage by enhancing intracellular antioxidant defense mechanisms such as Nrf2 and its target genes. Under physiological conditions, cytoplasmic Nrf2 is bound to Keap1 for the purpose of degradation [65]. However, UV radiation-induced oxidative stress was found to diminish Keap1 expression in skin keratinocytes and decrease formation of the Nrf2-Keap1-Cul3 complex [66], while this study showed that rutin prevented decreases in Keap1 expression after UVA and UVB irradiation. It was previously shown that rutin induces cellular defense genes by repressing Keap1-mediated inhibition of Nrf2 inhibition in vivo in liver tissue [67]. These effects on Keap1 can be attributed to the properties of rutin's polyphenolic component, quercetin, which may interact with Nrf2-binding sites in the Keap1 protein [67, 68]. The present study also showed that rutin attenuated UV-induced enhancement of KAP1 and p62 expression, inhibitors of Nrf2-Keap1-Cul3 complex formation. Unbound, active Nrf2 is translocated to the nucleus, where it binds to ARE elements in the DNA [15]. This interaction is facilitated by UV-induced reductions in the level of Bach1, which also binds to DNA sequences within the ARE elements [69]. Redox regulation of Bach1 is an alternative mechanism for inducing multiple ARE-dependent genes [70]. Treatment of cells with rutin both before and after UV exposure prevented oxidation of Bach1 cysteine residues, thereby enhancing Bach1 biological activity and consequently reducing HO-1 expression. Additionally, rutin induced a number of cellular antioxidants and phase II metabolic enzymes, including Cu/Zn-SOD, in HepG2 cells [71]. This observation may be associated with enhanced expression of p21, a factor that protects cells from oxidative stress through upregulation of the Nrf2 signaling pathway [72]. Flavonoids also exerted cytoprotective effects by enhancing p21 expression in colon cancer cells [73]. Additionally, changes in p21 expression may be connected with the changes in level of 4-HNE, which is involved in cell cycle progression [74], as well as with p53 activity, in UV-irradiated mouse fibroblasts lacking* p21* or* 53* genes [75]. Furthermore, rutin-mediated changes in p21 levels do not necessitate changes in p53 levels; a similar effect by flavones was observed previously in HT-29 cells [76]. It has also been shown that a decrease in KAP1 expression caused by gene knockdown leads to disruption in KAP1-mediated transcriptional repression of p21 in HEK293 cells [77]. These previous findings suggest that the p21 increases observed here might be the result of rutin-induced enhancement in KAP1 levels.

Nrf2 transcriptional activity is also dependent on its phosphorylation. Under physiological conditions, rutin enhances Nrf2 phosphorylation, while UV irradiation leads to its decrease. A possible mechanism for Nrf2 activation may be associated with the activity of quercetin, which may enhance the phosphorylation of JNK, p38, PI3K/Akt, and Nrf2 DNA-binding activity, which was shown previously for HepG2 cells [78]. It has also been suggested that rutin can enhance Nrf2 phosphorylation by increasing ERK activity in macrophages [79]. However, enhanced Nrf2 phosphorylation may also be associated with the increased anandamide or 2-AG levels observed in this study, while significant downregulation of cannabinoid receptor levels after rutin treatment of UV-irradiated fibroblasts may result in decreased signal transduction via the downregulation of protein phosphorylation. Nrf2 expression in rutin-treated, UV-irradiated fibroblasts was downregulated and positively correlated with expression of another transcription factor, NF*κ*B, the activation of which is also dependent on the actions of ROS and reactive aldehydes generated during lipid peroxidation [80].

### 4.4. Rutin Regulates Antioxidants Level and Activity

Rutin, by reducing Nrf2 expression, decreased UV radiation-induced increases in the levels and activities of antioxidant proteins including HO-1, GSH-Px, and GSSG-R. Moreover, by regulating the level of GSH, rutin facilitated the degradation of peroxides, including lipid peroxides, and effectively protected phospholipids from peroxidation. Previous in vivo studies have shown that rutin treatment significantly attenuates reductions in the levels and activities of GSH and GSH-dependent enzymes (GSH-Px and GSSG-R) in various rat models of disease [55]. Finally, rutin-mediated regulation of the redox balance in fibroblasts also prevented reductions in nonenzymatic antioxidants, including vitamins E and C, after UV irradiation.

### 4.5. Rutin Protects Phospholipids from Peroxidation

Rutin is one of the flavonoids that has shown the greatest ability to protect phospholipids from radical-mediated peroxidation [81] and enzymatic lipid oxidation via inhibition of cyclooxygenase-2 activity [82, 83]. Accordingly, we demonstrated that rutin decreases ROS generation and COX expression, thereby protecting fibroblast membrane phospholipids and proteins from UV radiation. The results of the present study confirmed that rutin prevents UV irradiation-induced, lipid radical-mediated peroxidation, as evidenced by reduced levels of reactive aldehydes (4-HNE and MDA) generated during oxidative fragmentation of phospholipid polyunsaturated fatty acids. Similar to other flavonoids, rutin exhibited partial lipophilic characteristics and may be partially localized on the surface of biomembranes. However, rutin is more hydrophilic than *α*-tocopherols and may therefore efficiently trap chain-initiating peroxyl radicals from the aqueous environment and cooperate with *α*-tocopherol to directly scavenge these species, which has been previously suggested from data generated using lymphoid cell lines [17, 84]. This presumption is reasonable because vitamin E levels in UV-irradiated fibroblasts were also increased after rutin treatment. Evidence to support the role of rutin in phase-two metabolism comes from studies of the compound's ability to protect erythrocyte membrane phospholipids from oxidative damage induced by* tert*-butyl hydroperoxide [85]. Additionally, rutin significantly reduced MDA formation after UV-induced lecithin peroxidation [86] and exerted protective activities in numerous biological systems under physiological conditions by increasing GSH levels and reducing MDA levels [53].

### 4.6. Rutin Protects Proteins from UV-Induced Oxidative Modifications

Rutin significantly reduced ROS and electrophilic reactive aldehyde generation resulting from UV exposure, thereby preventing reactions with the nucleophilic centers of amino acid residues and modification of their structures and functions. ROS primarily modifies aromatic protein residues, such as tryptophan and tyrosine, which was demonstrated following UVA and UVB irradiation in this study, while electrophilic aldehydes primarily modify cysteine and histidine residues by Michael addition and alter lysine structures via formation of Schiff base products [87]. The results of this study indicated that rutin prevents His-4-HNE adduct formation following UV irradiation. Moreover, rutin pretreatment attenuated nonspecific reactions of ROS and reactive aldehydes with protein amino acids, leading to increases in the levels of protein carbonyl groups after UV irradiation.

### 4.7. Rutin Protects Fibroblasts from UV-Induced Proapoptotic Actions

Rutin significantly protected fibroblasts from UV-induced apoptosis, particularly in response to UVA, through reduced caspase activation and cytochrome c release, as well as increased Bcl-2 expression. These data also suggested that inhibition of HO-1 expression, observed in this paper, might modulate rutin-mediated cell survival. Moreover, it was previously shown that rutin pretreatment significantly attenuates H_2_O_2_-induced apoptosis in HUVEC cells [88]. The antiapoptotic functions of rutin may synergize with its ability to protect DNA from oxidative damage, which has been shown for ischemia in rat brains [89]. Additionally, rutin treatment reduced the expression of p53, a protein involved in activation of DNA repair mechanisms and induction of apoptosis in response to DNA damage.

## 5. Conclusion

Rutin protects fibroblasts from UVA- and UVB-induced redox imbalance at protein and genes expression level. It also prevents changes in phospholipids metabolism leading to enhanced levels of electrophilic peroxidation products and decreased endocannabinoids levels and antiapoptotic activity. Thus, rutin is a promising compound that can protect the skin from the molecular consequences of ultraviolet radiation.

## Figures and Tables

**Table 1:** The activity of enzymatic (GSH-Px, GSSG-R, SOD, and TrxR) and the level of nonenzymatic (GSH, vitamin E, and Thx) antioxidants in fibroblasts after exposure to UVA [20 J/cm^2^], UVB radiation [200 mJ/cm^2^], and rutin [25 *µ*M] treatment, expressed as a percentage of the control cells value.

		Control	UVA	UVB
GSH-PX activity	Without rutin	100% ± 7%	153% ± 11%^x^	258% ± 12%^x^
	With rutin	90% ± 4%^x^	126% ± 7%^xa^	127% ± 9%^xa^

GSSG-R activity	Without rutin	100% ± 5%	198% ± 10%^x^	265% ± 13%^x^
	With rutin	129% ± 9%^xa^	171% ± 8%^xa^	267% ± 13%^x^

SOD activity	Without rutin	100% ± 6%	83% ± 4%^x^	54% ± 3%^x^
	With rutin	109% ± 6%	95% ± 4%^a^	83% ± 5%^xa^

TrxR activity	Without rutin	100% ± 5%	83% ± 4%^x^	74% ± 3%^x^
	With rutin	108% ± 6%	93% ± 4%	87% ± 5%^xa^

GSH level	Without rutin	109% ± 6%	64% ± 4%^a^	59% ± 5%^xa^
	With rutin	104% ± 5%	78% ± 3%^xa^	78% ± 4%^xa^

Vitamin E level	Without rutin	100% ± 6%	88% ± 4%^x^	74% ± 4%^x^
	With rutin	109% ± 5%	96% ± 4%	86% ± 4%^xa^

Trx level	Without rutin	100% ± 5%	81% ± 4%^x^	64% ± 4%^x^
	With rutin	104% ± 5%	97% ± 4%^a^	83% ± 4%^xa^

Mean values ± SD of five independent experiments are presented.

^x^Statistically significant differences versus control group, *p* < 0.05.

^a^Statistically significant differences versus group without rutin, *p* < 0.05.

**Table 2:** The level of oxidative modifications products of DNA, lipids, and proteins in fibroblasts after exposure to UVA [20 J/cm^2^], UVB radiation [200 mJ/cm^2^], and rutin [25 *µ*M] treatment and pretreatment, expressed as a percentage of the control cells value.

		Control	UVA	UVB
8-OHdG level	Without rutin	100% ± 5%	128% ± 11%^x^	167% ± 12%^x^
	With rutin	104% ± 6%	113% ± 7%^x^	147% ± 7%^xa^

4-HNE level	Without rutin	100% ± 5%	161% ± 10%^x^	132% ± 13%^x^
	With rutin	94% ± 6%	96% ± 5%^a^	90% ± 5%^a^

MDA level	Without rutin	100% ± 5%	150% ± 8%^x^	136% ± 7%^x^
	With rutin	95% ± 8%	108% ± 5%^a^	99% ± 5%^a^

Iso-prostaglandin F2*α* level	Without rutin	100% ± 5%	223% ± 11%^x^	248% ± 12%^x^
	With rutin	106% ± 7%	163% ± 9%^xa^	229% ± 12%^x^

4-HNE-protein adducts level	Without rutin	100% ± 6%	132% ± 7%^x^	150% ± 8%^x^
	With rutin	102% ± 5%	108% ± 5%^a^	129% ± 6%^xa^

Carbonyl groups level	Without rutin	100% ± 6%	153% ± 9%^x^	191% ± 9%^x^
	With rutin	110% ± 6%	145% ± 9%^x^	155% ± 8%^xa^
	With rutin pretreatment	108% ± 5%	118% ± 9%^xab^	129% ± 7%^xab^

Tryptophan level	Without rutin	100% ± 4%	80% ± 4%^x^	76% ± 3%^x^
	With rutin	97% ± 5%	86% ± 7%^x^	84% ± 4%^xa^

Tyrosine level	Without rutin	100% ± 5%	73% ± 4%^x^	77% ± 4%^x^
	With rutin	94% ± 4%	95% ± 5%^a^	92% ± 7%^a^

3Cl-tyrosine level	Without rutin	100% ± 6%	125% ± 7%^x^	159% ± 8%^x^
	With rutin	101% ± 5%	108% ± 5%^a^	109% ± 5%^a^

3NO_2_-tyrosine level	Without rutin	100% ± 4%	320% ± 16%^x^	339% ± 17%^x^
	With rutin	95% ± 5%	272% ± 14%^xa^	293% ± 15%^xa^

Dityrosine level	Without rutin	100% ± 7%	165% ± 8%^x^	149% ± 7%^x^
	With rutin	111% ± 5%	146% ± 7%^xa^	132% ± 7%^xa^

Mean values ± SD of five independent experiments are presented.

^x^Statistically significant differences versus control group, *p* < 0.05.

^a^Statistically significant differences versus group without rutin, *p* < 0.05.

^b^Statistically significant differences versus group without rutin pretreatment, *p* < 0.05.
